# Breast cancer knowledge in Lebanese females with positive family history

**DOI:** 10.1097/MD.0000000000032973

**Published:** 2023-02-17

**Authors:** Paul El Maouchi, Omar Fakhreddine, Abdel Hadi Shmoury, Mohamad El Zoghbi, Nathalie Chamseddine, Reine Abou Zeidane, Ghid Amhaz, Maya Charafeddine, Houry Kazarian, Hazem I. Assi

**Affiliations:** a Department of Internal Medicine, MedStar Health, Baltimore, MD; b Department of Internal Medicine, American University of Beirut Medical Center, Beirut, Lebanon; c Faculty of Medicine, American University of Beirut, Beirut, Lebanon; d Department of Obstetrics and Gynecology, American University of Beirut Medical Center, Beirut, Lebanon; e Department of Internal Medicine, Division of Hematology-Oncology, Naef K. Basile Cancer Institute, American University of Beirut Medical Center, Beirut, Lebanon.

**Keywords:** breast cancer, breast self-examination, screening

## Abstract

Breast cancer is the most commonly diagnosed cancer in women and the second leading cause of cancer-related death worldwide. Positive family history increases the likelihood of developing this disease. As late-stage presentation and poor survival rates are associated with a lack of knowledge about breast cancer and its screening methods, this study aimed to evaluate the knowledge of Lebanese women with first-degree relatives who were diagnosed with breast cancer. In this cross-sectional study, 200 women with a positive family history accompanying their relatives to oncology clinics or the infusion center at the American University of Beirut Medical Center, completed an online survey after institutional review board approval was granted. Demographic information and answers to questions related to breast cancer risk factors, warning signs, and screening techniques were collected and analyzed using descriptive statistics and chi-square tests. Eighty-one percent of the study participants agreed that a history of breast cancer is associated with a higher disease risk. The smaller portions were aware of other potential risk factors, such as hormone replacement therapy, alcohol consumption, late menopause, early menarche, and overweight and sedentary lifestyles. Also, 93% to 96.5% of the participants recognized breast self-examination and mammography as useful tools for early detection. Furthermore, younger participants who reached university level and were employed had more insights into breast cancer. Breast cancer knowledge and early diagnosis are key elements in preventing late presentations and reducing the associated morbidity and mortality. Further educational and awareness campaigns should be conducted in Lebanon to improve women knowledge of breast cancer.

## 1. Introduction

Breast cancer is the second most common cancer worldwide and the most frequently diagnosed cancer among females. The lifetime risk of developing breast cancer in every woman in the United States is approximately 12.4%, or 1 in 8 females.^[1]^ It is considered one of the leading causes of death from cancer with 198,000 deaths per year, which represents approximately 15.4% of all deaths in developed countries after lung cancer.^[2]^ Although this disease occurs worldwide, its incidence varies considerably between countries and regions, and with 4- to 5-fold variation. It is the highest in Europe and North America and the lowest in Asia.^[3]^ As for Lebanon, breast cancer is the most commonly diagnosed cancer among women, with an incidence of 94.5 per 100,000 females, and representing approximately 38.6% of all female cancers.^[4]^

Multiple factors are associated with an increased risk of breast cancer and are divided into hereditary and nonhereditary factors. It is well known that most hereditary cases are related to mutations in BRCA1 and BRCA2. Concerning nonhereditary factors, the most important contributing factors to breast cancer are female sex, aging, early menarche, late menopause, radiation exposure, and an unhealthy lifestyle such as smoking and obesity.^[5]^

Approximately a quarter of all breast cancer cases are related to a family history.^[6]^ In the United Kingdom, a cohort study of approximately 113,000 females found that having 1 first-degree relative with breast cancer had a 1.75-fold higher risk of developing this disease compared to females with healthy first-degree relatives, this risk increased by approximately 50% in females with 2 or more affected relatives.^[7]^

Breast cancer is classified according to the disease stage, including local, locally advanced, regionally advanced, and metastatic disease.^[8]^ Survival rates vary according to the stage of disease, whereas females with localized disease have a 98.9% 5-year survival rate, compared to 85.2% and 26.3% in regional and distant disease, respectively.^[9]^ These variations indicate the survival benefits of early detection. In addition, survival rates vary considerably between countries, where they are around 80% or greater in North America, Japan, and Australia; 60% in Brazil and Slovakia; and around 40% in Algeria.^[10]^ This significant decrease in survival among developing countries can be explained by the lack of early detection programs, leading to late-stage disease presentation.^[11]^

Early detection of breast cancer requires knowledge and awareness of early warning signs as well as screening methods. Breast cancer signs and symptoms can be classified into 3 main categories: breast lumps, nonlump breast symptoms such as breast pain, breast skin or shape changes, and nipple abnormalities, and nonbreast symptoms such as fatigue, weight loss, neck lumps, and axillary symptoms.^[12]^ A cross-sectional study conducted in the United Arab Emirates assessing awareness of breast cancer and breast cancer self-examination among female students at the University of Sharjah in 2019 showed that among the warning signs and symptoms, breast lumps (80.1%), change in breast size and shape (74.7%), and pain in the breast or armpit (73.3%) were the most commonly identified correctly, whereas nipple abnormalities (37.8%) and arm swelling were the least identified (26.1%).^[13]^ The screening methods include breast self-examination, clinical breast examination, and mammography. Among these 3 methods, only mammography decreased mortality by 15% in women aged 39 to 49.^[14]^ Previous studies conducted in developing countries have shown that only a small percentage of women undergo breast self-examination and periodic mammography, which could be attributed to a lack of knowledge on how to perform breast self-examination or fear of discovering malignancy.^[15]^

Lack of knowledge about breast cancer may lead to late-stage presentation and lower survival rates. Females, especially those with a high risk, must be capable of identifying breast cancer risk factors, early signs and symptoms and be aware of available screening methods.^[16]^ Therefore, conducting a study to assess the level of knowledge in females with a positive family history is essential to promote awareness campaigns aimed at decreasing the disease burden and mortality rates.

## 2. Materials and methods

### 2.1. Selection and description of participants

#### 2.1.1. Inclusion criteria.

• Women aged 18 and above.
• Women with a family history of breast cancer in a first-degree relative.

#### 2.1.2. Exclusion criteria.

• Women diagnosed with breast cancer.
• Women under the age of 18.
• Women with no family history of breast cancer in their first-degree relatives.

### 2.2. Technical information

#### 2.2.1. Study design.

This cross-sectional study analyzed data obtained from females who accompanied or visited their first-degree relatives at oncology clinics and the infusion center at the American University of Beirut Medical Center to assess their level of knowledge about breast cancer risk factors, early warning signs, and screening approaches. The invitation script, consent form, and Lime Survey questionnaire were provided online to individuals who met the inclusion criteria.

#### 2.2.2. Sample size.

The target sample size was 200 female participants. A total of 412 online survey invitations were sent until the target number of 200 responses was reached, with a response rate of 48.5%.

#### 2.2.3. Instrument.

The questionnaire is adapted from the breast cancer awareness measure tool version 2.

This questionnaire includes data regarding: sociodemographic characteristics of participants with their medical and family history; knowledge about breast cancer risk factors; knowledge about early signs of breast cancer; knowledge and attitudes towards breast cancer screening methods.

### 2.3. Statistics

Data were collected through the Lime Survey and analyzed using SPSS IBM version 27.0. Descriptive statistics (frequencies and percentages) were used to describe the demographic characteristics, knowledge of breast cancer risk factors, early signs, and screening methods. A chi-square test was used to correlate the demographic characteristics of the participants with their breast cancer knowledge. Statistical significance was set at *P* < .05.

### 2.4. Ethical consideration

This study was conducted in accordance with the declaration of Helsinki guidelines. This study was approved by the institutional review board of the American University of Beirut (protocol code SBS-2020-0410, date of approval: April 13, 2021).

## 3. Results

Our sample consisted of 200 female participants who had completed an online survey. The demographic characteristics subsection of the survey was answered by 190 participants; the results are summarized in Table 1. The majority of our sample was 18 to 44 years old (68.9%), lived in Beirut (41.6%), married or previously married (62.1%), had reached university level (83.2%), and was employed (58.4%).

**Table 1:** Demographic characteristics of the sample population.

Demographic characteristics (N = 190)	Frequency	Percentage
Age		
18–44	131	68.9%
45 and above	59	31.1%
Area of residency		
Beirut	79	41.6%
Mount Lebanon	49	25.8%
Other areas	62	32.6%
Marital status		
Single	72	37.9%
Married/previously married	118	62.1%
Education level		
Primary/secondary school	32	16.8%
University and above	158	83.2%
Occupation status		
Employed	111	58.4%
Unemployed	79	41.6%

Table 2 summarizes the responses to the questionnaire items related to risk factors and warning signs of breast cancer. There was a wide agreement that having a history of breast cancer (81.0%) or having a close relative with breast cancer (84.0%) were both risk factors for breast cancer. However, the participants were less knowledgeable about other risk factors, such as the use of hormone replacement therapy (50.0%), alcohol consumption (28.5%), being overweight (34.0%), having children later in life (23.0%), suboptimal physical activity (8.5%), and early menarche or late menopause (15.5% and 17.0%, respectively). Table 2 also shows that the majority of the sample was well-informed about breast cancer warning signs.

**Table 2:** Answers to the questionnaire items related to risk factors and warning signs of breast cancer.

Questionnaire items (N = 200)	Responses	
**Risk factors**	**Agree**	**Disagree**
Having a past history of breast cancer	162 (81.0%)	38 (19.0%)
Using hormone replacement therapy (HRT)	100 (50.0%)	100 (50.0%)
Drinking more than 1 drink of alcohol a day	57 (28.5%)	143 (71.5%)
Being overweight (BMI over 25)	68 (34.0%)	132 (66.0%)
Having a close relative with breast cancer	168 (84.0%)	32 (16.0%)
Having children later in late or not at all	46 (23.0%)	154 (77.0%)
Starting your period at an early age (before the age of 12)	31 (15.5%)	169 (84.5%)
Having late menopause (after the age 55)	34 (17.0%)	166 (83.0%)
Doing suboptimal physical activity	17 (8.5%)	183 (91.5%)
**Warning signs**	**Yes**	**No**
Change in the position of the nipple	152 (76.0%)	48 (24.0%)
Pulling in the nipple	119 (59.5%)	81 (40.5%)
Pain in one of the breasts or armpits	137 (68.5%)	63 (31.5%)
Puckering or dimpling of the breast skin	129 (64.5%)	71 (35.5%)
Discharge or bleeding from the nipple	160 (80.0%)	40 (20.0%)
Lump or thickening in the breast	184 (92.0%)	16 (8.0%)
Lump or thickening under the armpit	176 (88.0%)	24 (12.0%)
Nipple rash	103 (51.5%)	97 (48.5%)
Redness of the breast skin	121 (60.5%)	79 (39.5%)
Changes in the size of the breast or nipple	145 (72.5%)	55 (27.5%)
Changes in the shape of the breast or nipple	159 (79.5%)	41 (20.5%)

BMI = body mass index.

Table 3 summarizes the answers to the questionnaire items related to breast cancer screening techniques. Of the participants, 93% had previously heard of breast self-examination (BSE), and most were taught how to perform BSE by a healthcare professional. In total, 58.0% correctly answered that BSE should be started at 20 years of age and performed on a monthly basis. The best timing for BSE was 1 week after menses (62.0% correct). The majority of the sample (82.5%) had heard of clinical breast examination (CBE) and 60.5% correctly answered that it should be performed on a yearly basis. Almost 9 out of 10 participants had previously heard of mammography and believed that it should be performed every 1 to 2 years, as it is a useful tool for the detection of breast cancer. However, only 34.0% thought that it should be started after the age of 40. Approximately half of our sample had previously undergone mammography, while the majority of the other half was not old enough to be eligible.

**Table 3:** Answers to the questionnaire items related to breast cancer screening techniques.

Questionnaire items (N = 200)	Frequency	Percentage
Have you heard of breast self-examination (BSE)?		
Yes	186	93.0%
No	14	7.0%
Have you been taught how to do BSE?		
Yes	139	69.5%
No	61	30.5%
If yes to the question, who taught you how to do BSE? (n = 139)		
Healthcare worker	92	66.2%
Family or friend	16	11.5%
Awareness campaigns	31	22.3%
At what age do you think BSE should be started?		
20 yr	116	58.0%
30 yr or above	65	32.5%
I don’t know	19	9.5%
How often do you think BSE should be done?		
Daily or weekly	43	21.5%
Monthly	116	58.0%
Yearly	18	9.0%
I don’t know	23	11.5%
What is the best time to do BSE?		
During menstrual flow	3	1.5%
A week after period	124	62.0%
During breast feeding	2	1.0%
I don’t know	71	35.5%
What are the benefits of BSE?		
To be familiar with the breast texture	4	2.0%
Early detection of breast cancer	76	38.0%
Detection of abnormal changes in the breast	110	55.0%
I don’t know	10	5.0%
Have you heard of clinical breast examination (CBE)?		
Yes	165	82.5%
No	35	17.5%
How often do you think CBE should be done?		
Monthly	5	2.5%
Yearly	121	60.5%
When an abnormality is found on BSE	40	20.0%
I don’t know	34	17.0%
Have you ever heard of mammography?		
Yes	193	96.5%
No	7	3.5%
Is mammography is useful tool for detection of breast cancer?		
Yes	183	91.5%
No	7	3.5%
I don’t know	10	5.0%
At what age should mammography be started in Lebanon?		
Before 40	114	57.0%
After 40	68	34.0%
I don’t know	18	9.0%
How often should mammography be done?		
Every 1–2 yr	179	89.5%
Every 3–5 yr	9	4.5%
I don’t know	12	6.0%
Have you ever done a mammography?		
Yes	95	47.5%
No	105	52.5%
If no to the question above, why not? (n = 105)		
Not old enough	73	69.5%
No reason or other reason	32	30.5%

BSE = breast self-examination, CBE = clinical breast examination.

Table 4 shows the distribution of correct responses to the questionnaire items (risk factors, warning signs, and screening techniques), according to the demographic characteristics of the sample. Only statistically significant associations are presented in Table 4 (*P* < .05). In summary, participants who were 18 to 44 years of age, married, reached university level, employed were most likely to correctly answer questions related to risk factors, warning signs, and breast cancer screening techniques.

**Table 4:** Distribution of responses according to the demographic characteristics of the sample.

Questionnaire items	Demographic characteristics (N = 190)		*P* value
	**Age**		
	**18–44**	**45 and above**	
Doing suboptimal physical activity	14 (10.7%)	1 (1.7%)	.033
Lump or thickening under the armpit	120 (91.6%)	46 (78.0%)	.009
Starting BSE at 20 yr of age	85 (64.9%)	26 (44.1%)	.024
	**Marital status**		
	**Single**	**Married**	
Changes in the size of the breast or nipple	43 (59.7%)	92 (78.0%)	.007
	**Education level**		
	**School**	**University**	
Having a past history of breast cancer	19 (59.4%)	135 (85.4%)	.001
Having a close relative with breast cancer	22 (68.8%)	139 (88.0%)	.006
Lump or thickening in the breast	26 (81.3%)	148 (93.7%)	.021
Starting BSE at 20 yr of age	11 (34.4%)	100 (63.3%)	.007
	**Occupation status**		
	**Employed**	**Unemployed**	
Discharge or bleeding from the nipple	94 (84.7%)	56 (70.9%)	.021
Doing BSE monthly	74 (66.7%)	38 (48.1%)	.007
Doing mammography every 1 to 2 yr	106 (95.5%)	64 (81.0%)	.005

BSE = breast self-examination.

## 4. Discussion

To the best of our knowledge, this is the first cross-sectional study to assess breast cancer knowledge as it pertains to risk factors, early warning signs, and awareness and attitudes towards screening methods among Lebanese females with a positive family history.

Concerning breast cancer risk factors, most participants acknowledged a history of breast cancer (81%) and had a close relative with breast cancer (84%) as a well-established risk factor for developing breast cancer. A relatively small number of patients were aware that hormone replacement therapy (58%) increased the risk of breast cancer. However, only a minority of participants were aware that alcohol use (28.5%), being overweight (34%), early menarche (15.5%), late menopause (17%), and a sedentary lifestyle (8.5%) were potential risk factors. These findings are congruent with the findings of a previous study assessing breast cancer awareness among 200 Saudi females in Jeddah, which revealed that most of the participants were aware of family history and hormone replacement as risk factors for breast cancer.^[17]^ Similarly, insufficient awareness regarding other risk factors such as obesity, early menarche, and late menopause has been reported in a study by Al-Dubai et al^[18]^ that assessed awareness and knowledge of breast cancer and mammography among Malaysian females. These results suggest the need for educational and awareness programs on breast cancer risk factors.

Concerning awareness of the early warning signs of breast cancer, a lump or thickening in the breast was the most commonly identified sign of breast cancer (92%), and followed by a lump or thickening in the armpit (88%). The majority of participants were also aware that nipple discharge or bleeding (80%), change in the position of the nipple (76%), change in the size (72.5%), and change in the shape of the breast or nipple were early signs of disease. Nipple rash (51.5%) and nipple pulling (59.5) were less commonly recognized as early signs of breast cancer. These findings mirror the results reported by Grunfeld et al in their study on women knowledge and beliefs regarding breast cancer, where the majority of participants recognized a painless lump as a symptom of breast cancer, and less than half of the surveyed women were aware that skin dimpling and nipple retraction or eczema were early signs of breast cancer.^[19]^

With regard to awareness of breast self-examination, the majority (93%) of participants had heard of BSE and 69.5% had been taught by a healthcare professional how to perform BSE. More than half of the participants were aware of the frequency (58%), best time (62%), appropriate age (58%), and benefits of BSE. These results suggest a better knowledge of BSE in the Lebanese population than that reported in many previous studies in Jordan and Saudi Arabia.^[20]^

There is still some disagreement regarding the use of CBE as a screening tool; some societies, such as the American cancer society, do not recommend it, whereas the United States national comprehensive cancer network is still considering CBE as part of the guidelines.^[21]^ In our study, the majority of the participants (82.5%) had heard about CBE and more than half (60.5%) answered correctly about its frequency, which was in agreement with what had been reported in a previous study from Jordan, which showed that more than half of the participants correctly reported the frequency of CBE.^[22]^

The majority of participants in this study had heard about mammography (96.5%), showed a positive attitude towards its importance as a screening tool for breast cancer (91.5%), and answered correctly when asked how often it should be performed (89.5%). However, less than half of the participants had undergone mammographic examination (47.5%) and acknowledged the appropriate starting age for screening (34%). When asked about the reasons for not performing a mammogram, variable excuses were cited, and with not being in an adequate age group being the most reported excuse (69.5%). Our findings are similar to those of a study conducted among Korean American women which showed a discrepancy between the awareness of the importance of breast cancer screening and the actual practice of screening.^[23]^

In this study, knowledge regarding breast cancer risk factors, early signs, screening techniques was affected by multiple variables, including age, marital status, level of education, and occupational status. This shows that participants with a higher level of education had a better knowledge of breast cancer, as it pertains to risk factors, early signs, screening techniques, and which is consistent with what has been reported in Turkey.^[24]^ This study also showed that younger females (18–44 years) had better knowledge of breast cancer than older women (45 years and above), which contradicts the findings of Amin et al reported among Saudi women.^[25]^ In addition, married and employed women had significantly better knowledge than others. This is in agreement with what has been reported by Alam in Saudi Arabia and Taher in Iraq.^[26,27]^

An apparent limitation of this study is that the questionnaire was self-administered, which may have increased the risk of information bias. In addition, the participants were recruited from a single medical center, which makes it difficult to generalize the study findings.

## 5. Conclusion

Knowledge of breast cancer among females with a positive history is instrumental in preventing delayed presentation, which further decreases the rates of morbidity and mortality from breast cancer. Most of our participants failed to recognize alcohol consumption, being overweight, having a sedentary lifestyle, early menarche, or late menopause as risk factors for breast cancer. Women who were older, single, unemployed, and had lower educational levels had worse knowledge of breast cancer. This is important for implementing future educational and awareness campaigns targeting the subpopulation of females that lack the most knowledge.

## Author contributions

**Conceptualization:** Hazem Assi.

**Data curation:** Paul El Maouchi.

**Formal analysis:** Nathalie Chamseddine, Maya Charafeddine, Houry Kazarian.

**Project administration:** Paul El Maouchi, Houry Kazarian.

**Supervision:** Hazem Assi.

**Validation:** Nathalie Chamseddine.

**Writing – original draft:** Paul El Maouchi, Omar Fakhreddine, Abdel Hadi Shmoury, Mohamad El Zoghbi.

**Writing – review & editing:** Reine Abou Zeidane, Ghid Amhaz.
